# A high-throughput customized cytokinome screen of colon cancer cell responses to small-molecule oncology drugs

**DOI:** 10.18632/oncotarget.28079

**Published:** 2021-09-28

**Authors:** Kelsey E. Huntington, Anna Louie, Lanlan Zhou, Wafik S. El-Deiry

**Affiliations:** ^1^Laboratory of Translational Oncology and Experimental Cancer Therapeutics, Warren Alpert Medical School, Brown University, Providence, RI 02912, USA; ^2^The Joint Program in Cancer Biology, Brown University and Lifespan Health System, Providence, RI 02912, USA; ^3^Cancer Center at Brown University, Warren Alpert Medical School, Brown University, Providence, RI 02912, USA; ^4^Department of Pathology and Laboratory Medicine, Warren Alpert Medical School, Brown University, Providence, RI 02912, USA; ^5^Pathobiology Graduate Program, Brown University, Providence, RI 02912, USA; ^6^Department of Surgery, Brown University, Lifespan Health System and Warren, Alpert Medical School, Brown University, Providence, RI 02912, USA

**Keywords:** cytokine profiling, inflammatory response, chemokine, growth factor, immune profiling

## Abstract

Inflammatory cytokines, chemokines, and growth factors are molecular messengers that circulate and have the capability to modify the tumor microenvironment and impact therapeutic response. The characterization of soluble mediators as biomarkers for diagnosis and prognosis is of interest in oncology. We utilize the cytokinome to characterize the response of colorectal tumor cell lines to selected small-molecules in oncology as a proof-of-concept dataset with immunomodulatory analyte heat map rankings for drug and cell line combinations. We observed overall trends in drug class effects with MEK-, BRAF-, PARP-inhibitors, and Imipridones in cytokine, chemokine, and growth factor responses that may help guide therapy selection. MEK-inhibitor treatment downregulated analytes VEGF, CXCL9/MIG, and IL-8/CXCL8 and upregulated CXCL14/BRAK, Prolactin, and CCL5/RANTES. BRAF-inhibitor treatment downregulated VEGF and IL-8/CXCL8, while increasing soluble TRAIL-R2. Treatment with PARP-inhibitors decreased CXCL9/MIG, IL-8/CXCL8, CCL3/MIP-1 alpha, VEGF, and CXCL14/BRAK, while treatment increased soluble TRAIL-R2 and prolactin. Treatment with Imipridones decreased CCL3/MIP-1 alpha, VEGF, CXCL14/BRAK, IL-8/CXCL8, and Prolactin and increased CXCL5/ENA-78. We also observed differential responses to therapeutics depending on the mutational profile of the cell line. In the future, a similar but larger dataset may be utilized in the clinic to aid in the prediction of patient response to immunomodulatory therapies based on tumor genotype.

## INTRODUCTION

Cytokines, chemokines, and growth factors are all molecular messengers of the immune system that impact tumor behavior and host response. Cytokines are either secreted or membrane-bound proteins that regulate cellular signaling and can be categorized as pro- or anti-inflammatory. Chemokines are proteins that mediate chemotaxis in nearby cells and play an important role in the recruitment of either immunosuppressive or immunostimulatory cell types to the tumor microenvironment. Differential expression of chemokines regulates the selective migration of myriad cell types [1]. Growth factors are usually secreted proteins or steroid hormones that promote cell proliferation and differentiation. Each of these categories of immunomodulating agents are produced by both tumor and immune cells, among other cell types, and can impact therapeutic response [2, 3].

The characterization of these soluble mediators as biomarkers of both diagnosis and prognosis is a rapidly evolving topic in cancer research and clinical oncology. Biomarkers have been correlated with clinical outcome in several different tumor types including colorectal cancer (CRC) [4–6]. In CRC, differentially expressed plasma or serum cytokines represent potential biomarkers for diagnosis and prognosis. Yamaguchi et al. found that the levels of cytokines in plasma varied significantly between patients with CRC and control subjects [4]. However, cytokine signaling is highly pleotropic with one cytokine producing diverse and sometimes opposing effects depending on the signaling context [2]. Moreover, cytokine signaling is characterized by a high degree of redundancy where discrete cytokines produce the same functional effects [7]. The combination of pleotropic and redundant outcomes in response to a particular cytokine makes therapeutic manipulation challenging. Furthermore, there exists a degree of heterogeneity in the prognostic value of cytokines, with some showing opposing correlations in response to therapy across multiple tumor types [5, 8].

## RESULTS

### Cytokinome profiling of human colorectal cancer cell lines with diverse mutations using a high-throughput custom multiplexed analyte panel

Cell lines that represent diverse mutational backgrounds in tumor suppressor or oncogene drivers such as TP53, KRAS, BRAF, PIK3CA, APC, TRK, CTNNB1, BRCA2, TGFB2, and PTEN were selected for the analyses reported (Table 1). The three cell lines HCT-116, HT-29, and KM12C were included because they represent varied mutational profiles and were predicted to respond differently to differing therapeutic mechanisms of action. Importantly, we included both microsatellite stable (MSS) and microsatellite instability positive (MSI+) cell lines to observe how microsatellite status impacts response to small-molecule treatment, which has implications in combination with checkpoint blockade therapies.

**Table 1:** Mutational background of selected colon cancer cell lines

CRC Cell Line Name	Species	MSI/MSS status	TP53	HRAS	NRAS	KRAS	BRAF	PIK3CA	PTEN expression	APC	TRK	CTNNB1	ACVR2A	BRCA2	TGFBR2
**HCT-116**	human	MSI	WT	WT	WT	MT	WT	MT	positive	WT	WT	MT	MT	MT	WT
**HT-29**	human	MSS	MT	WT	WT	WT	MT	MT	positive	MT	WT	WT	WT	WT	MT
**KM12C**	human	MSI	MT	WT	WT	WT	WT	WT	null	MT	MT	UN	MT	MT	MT

Abbreviation: WT: wild type; MT: mutant; UN: unknown. HCT-116, HT-29, and KM12C colon cancer cell line mutational backgrounds.

**Figure 1:**
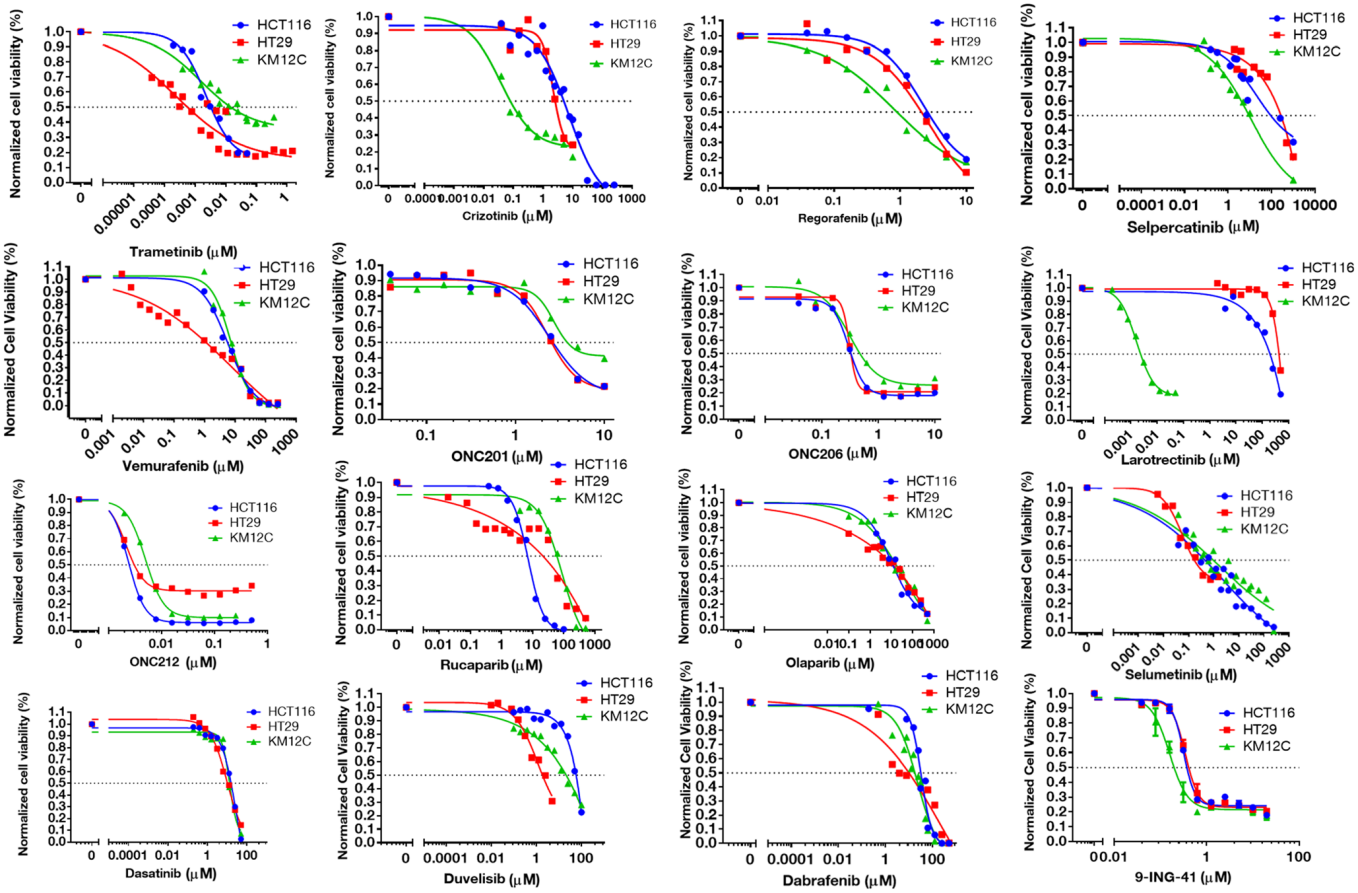
IC-50 curves for selected small-molecules in HCT-116, HT-29, and KM12C cell lines. Cell viability curves post 72-hour treatment were graphed in GraphPad and used to determine IC-50 values.

Selected oncologic small-molecules with distinct mechanisms of action encompassed several classes of drugs such as PARP-, MEK-, and BRAF-inhibitors, among others (Table 2). We primarily selected FDA-approved small-molecules, but also included several experimental small-molecules in oncology that target commonly dysregulated pathways in cancer. We were especially interested in the results of the experimental drugs that are either currently in clinical trials or are planned for clinical trials in the near future (such as GSK-3 inhibitor 9-ING-41, and Imipridones ONC201, ONC206, and ONC212). The selected cell lines displayed a range of susceptibility to the drug panel (Figure 1, Supplementary Table 2).

**Table 2:** Drug classes of selected small-molecule drugs

Drug	Class
Trametinib	MEK1/2 inhibitor
Crizotinib	Tyrosine kinase inhibitor
Larotrectinib	TRK inhibitor
Selpercatinib	RET inhibitor
Vemurafenib	BRAF inhibitor
Regorafenib	Tyrosine kinase inhibitor
ONC201	Imipridone
ONC206	Imipridone
ONC212	Imipridone
Rucaparib	PARP inhibitor
Olaparib	PARP inhibitor
Selumetinib	MEK inhibitor
Dabrafenib	BRAF inhibitor
Dasatinib	Tyrosine kinase inhibitor
Duvelisib	PI3K inhibitor
9-ING-41	GSK-3 inhibitor

Drug name is listed in the first column and drug class is listed in the second column.

**Figure 2:**
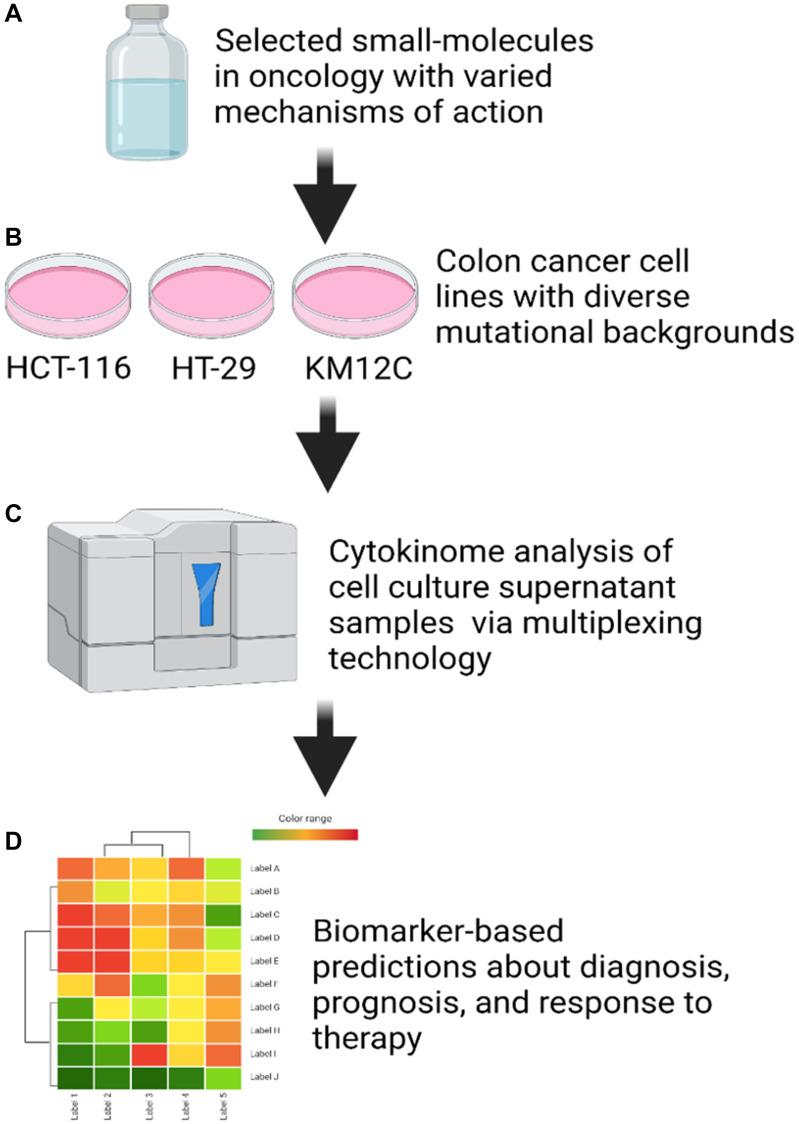
Cell culture supernatant cytokinome analysis workflow. (**A**) Small-molecules in oncology were selected to provide varied mechanisms of action. (**B**) Three colon cancer cell lines (HCT-116, HT-29, and KM12C) were selected based on differing mutational backgrounds in key tumor suppressor genes. (**C**) Cytokinome analysis was performed on cell culture supernatant samples after 48 hours of treatment using a Luminex 200. (**D**) Biomarker-based predictions about diagnosis, prognosis, and response to therapy were made based on heat maps generated from linear regression analysis of the data.

We designed a high-throughput custom multiplex cytokine, chemokine, and growth factor profiling panel based on both pro- and anti-inflammatory markers, as well as cytokines and chemokines involved in the recruitment and activation of immune cells such as natural killer (NK) and T cells. Cell lines were treated with the drug panel and cell culture supernatants were analyzed using Luminex 200 technology (Figure 2). Cell lines were treated at differing concentrations (IC-10, IC-30, IC-50, IC-70, and IC-90) to determine dose-response effects for each small-molecule. It is important to emphasize that the panel was designed to analyze soluble factors that are secreted or shed by tumor cells post-treatment with drug.

To analyze these results, we graphed dose-response values for each drug and cell line combination and calculated the linear regression. To generate heat maps that rank analytes for each drug and cell line combination from most-downregulated to most up-regulated, we utilized the slope of the linear regression. We then examined the top five most up- and down-regulated analytes from each heat map (Supplementary Table 1).

**Figure 3:**
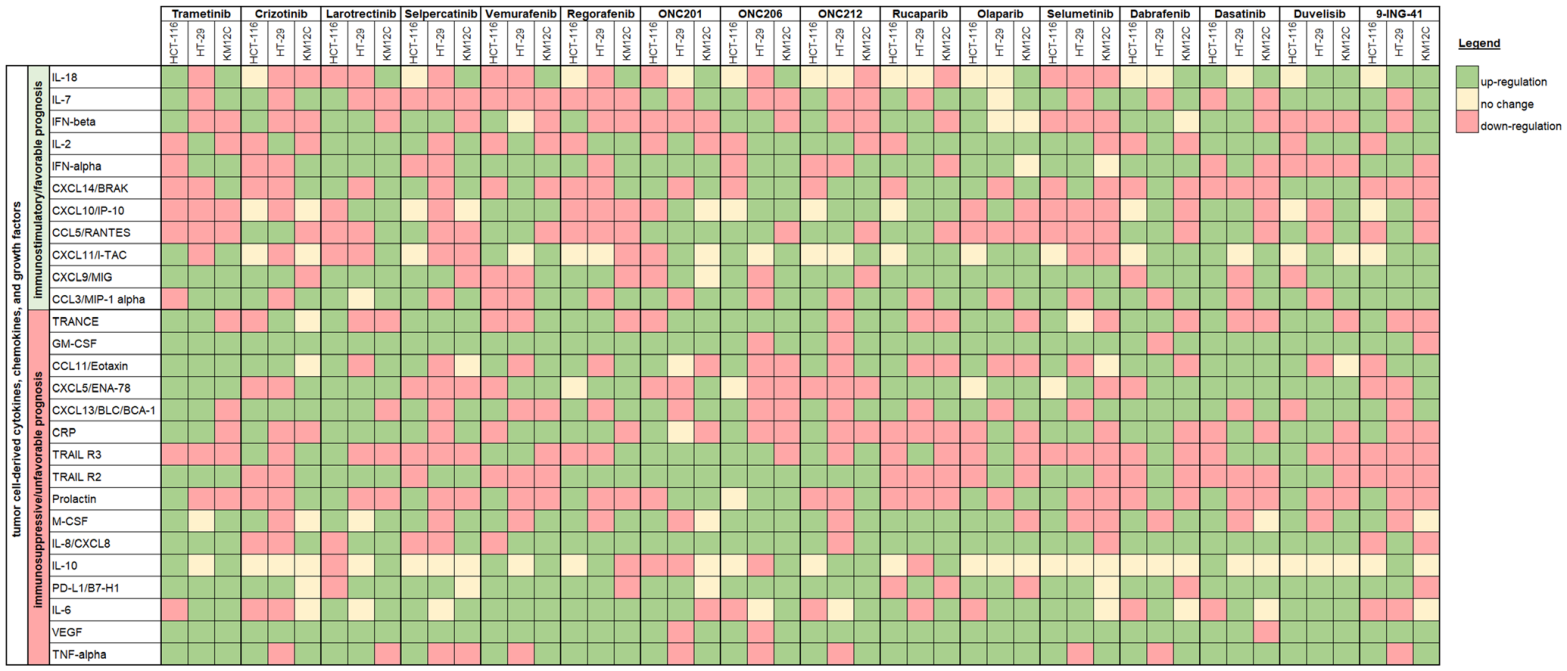
Immune synergy heat map showing cell line changes in cytokine, chemokine, and growth factor profiles in response to therapeutic treatment. Cytokines, chemokine, and growth factors are grouped into one of two categories: (1) analytes that are correlated with immunosuppression or unfavorable prognosis or (2) analytes that are correlated with immunostimulation or favorable prognosis in the context of CRC. The heat map is based on the slope of the linear regression where green indicates upregulation, yellow indicates no change, and red indicates downregulation post-treatment.

To better visualize the data, we created a summary heatmap that grouped cytokines, chemokines, and growth factors into two categories: (1) analytes that are correlated with immunosuppression or unfavorable prognosis [6, 9–18] or (2) analytes that are correlated with immunostimulation or favorable prognosis in the context of CRC, specifically [19–26] (Figure 3). When designing these two groups, we focused on the implications of a particular analyte when colon cancer cell-derived.

### Class effects on cytokine, chemokine, and growth factor profiles were observed across multiple human colorectal cancer cell lines

**Figure 4:**
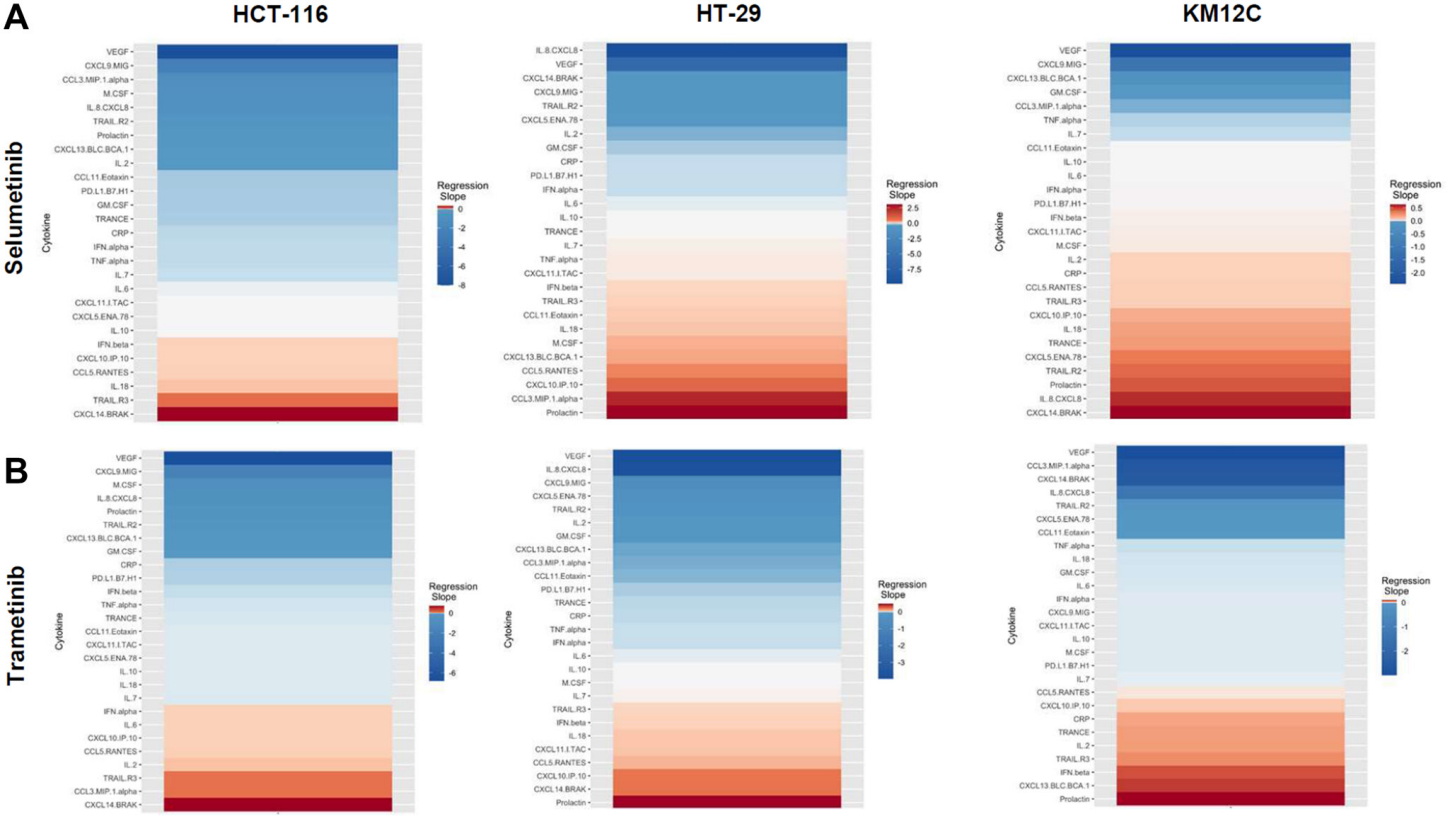
Heatmaps displaying regression slopes of cytokine profiles for MEK inhibitors. (**A**) Heat maps based on regression slopes for HCT-116, HT-29, and KM12C after 48-hour treatment of increasing doses of Selumetinib or (**B**) Trametinib.

**Figure 5:**
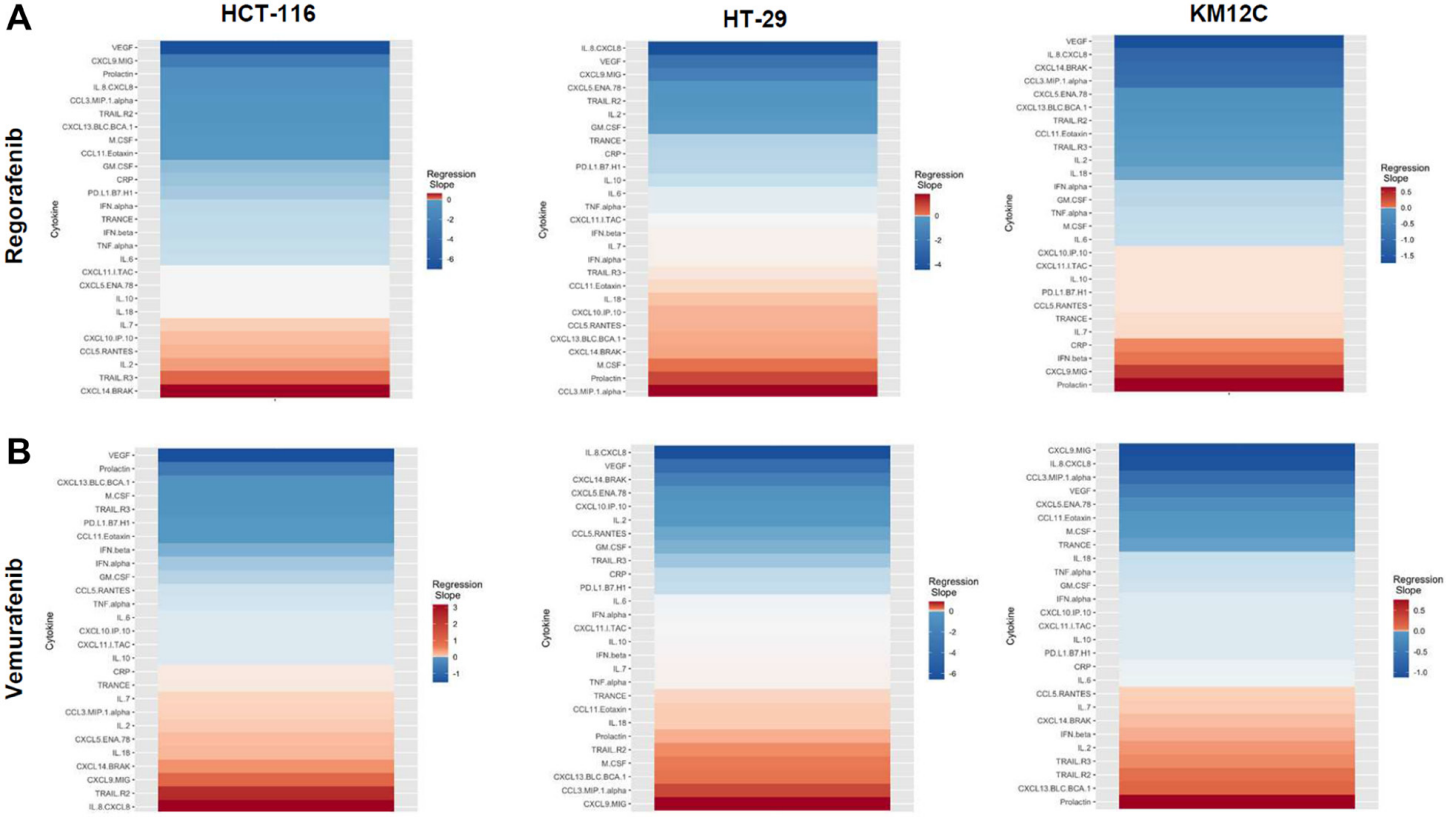
Heatmaps displaying regression slopes of cytokine profiles for BRAF inhibitors. (**A**) Heat maps based on regression slopes for HCT-116, HT-29, and KM12C after 48-hour treatment of increasing doses of Regorafenib or (**B**) Vemurafenib.

**Figure 6:**
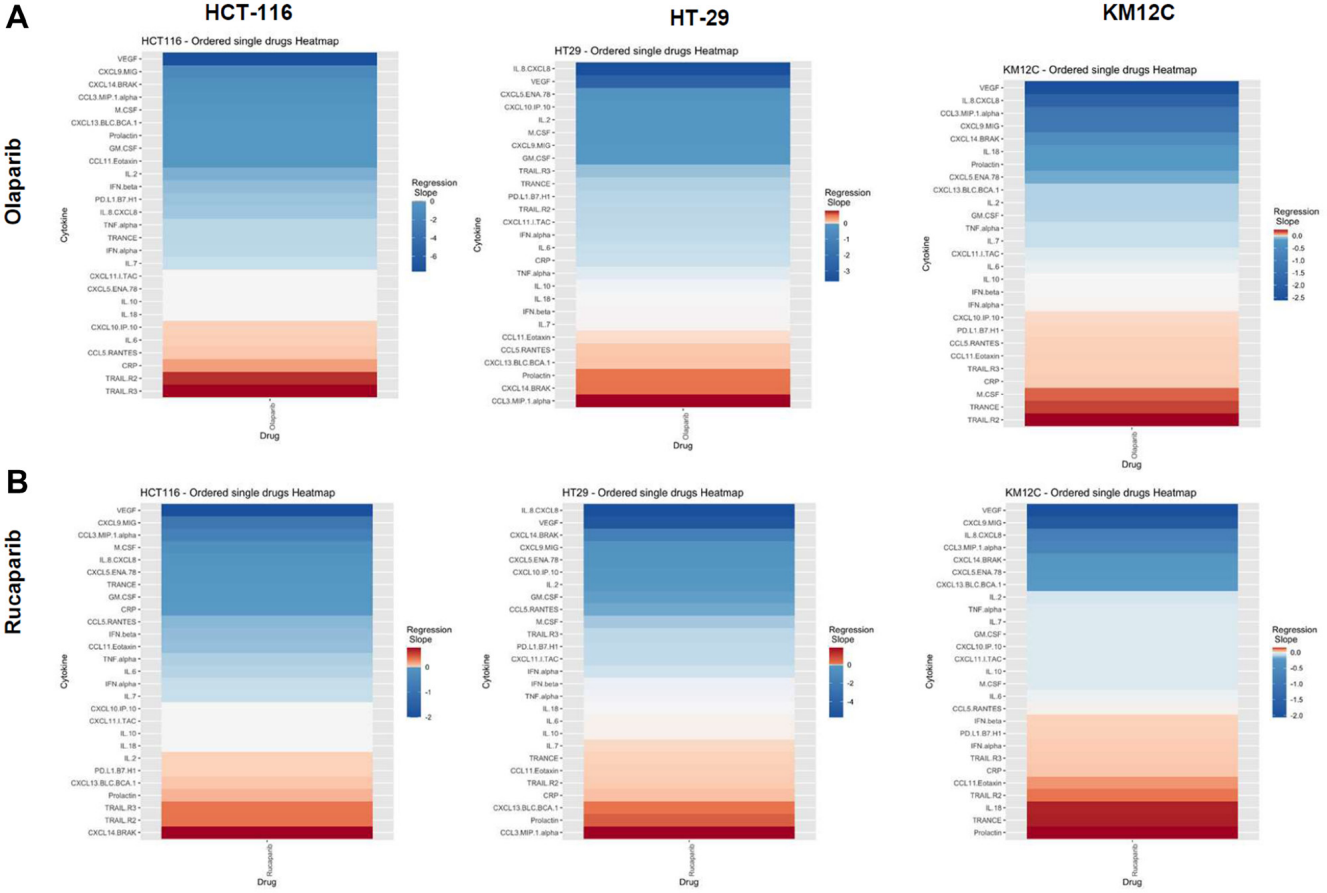
Heatmaps displaying regression slopes of cytokine profiles for PARP inhibitors. (**A**) Heat maps based on regression slopes for HCT-116, HT-29, and KM12C after 48-hour treatment of increasing doses of Olaparib or (**B**) Rucaparib.

**Figure 7:**
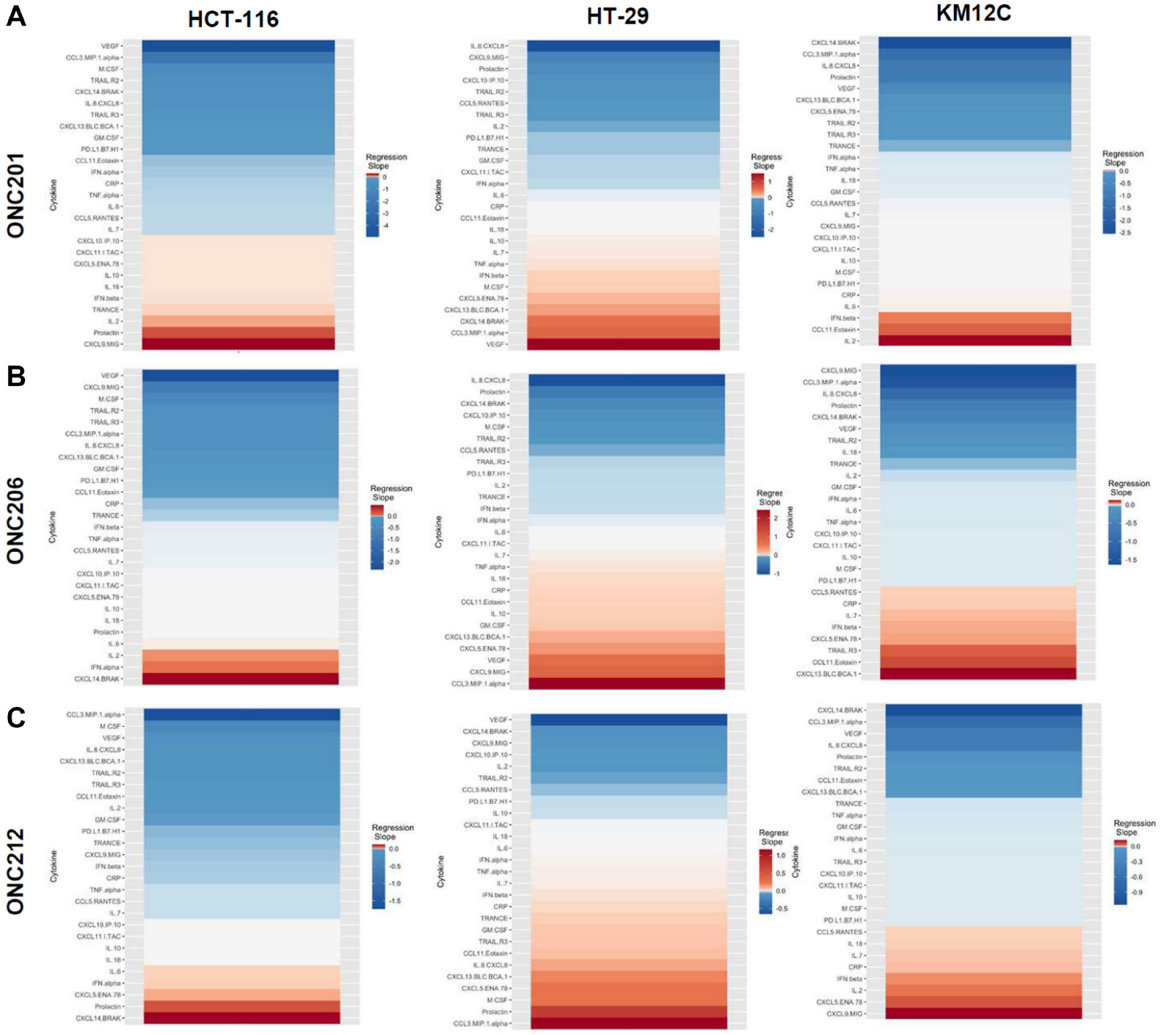
Heatmaps displaying regression slopes of cytokine profiles for Imipridones. (**A**) Heat maps based on regression slopes for HCT-116, HT-29, and KM12C after 48-hour treatment of increasing doses of ONC201, (**B**) ONC206, or (**C**) ONC212.

There were several classes of drugs of which we tested more than one inhibitor and observed class effects. Drug classes where we tested multiple compounds included MEK inhibitors, BRAF inhibitors, PARP inhibitors, and Imipridones. For MEK inhibitors, we tested both Trametinib and Selumetinib, and saw similarities in the top five most-downregulated analytes in response to both drugs across all three cell lines (6 groups total) (Figure 4). We saw decreases in VEGF (6 out of 6), CXCL9/MIG (5 out of 6), and IL-8/CXCL8 (5 out of 6). The analytes that most notably increased after treatment with MEK inhibitors in all cell lines were CXCL14/BRAK (4 out of 6), Prolactin (4 out of 6), and CCL5/RANTES (4 out of 6). Next, for BRAF inhibitors Dabrafenib and Vemurafenib we again saw similar trends in response across all cell lines tested (Figure 5). We observed decreases in VEGF (6 out of 6), and IL-8/CXCL8 (5 out of 6). By contrast, we observed increases in soluble TRAIL-R2 (sTRAIL-R2) (4 out of 6). Next, PARP inhibitors included Olaparib and Rucaparib and once again we observed notable decreases in VEGF (6 out of 6), CXCL9 (5 out of 6), IL-8 (5 out of 6), CCL3/MIP-1 alpha (4 out of 6), and CXCL14/BRAK (4 out of 6) (Figure 6). The analytes that increased as a class effect included sTRAIL-R2 (5 out of 6), and Prolactin (4 out of 6). Lastly, for Imipridones we tested three different compounds including ONC201, ONC206, and ONC212 (9 groups) (Figure 7). We saw notable decreases in VEGF (6 out of 9), CCL3/MIP-1 alpha (5 out of 9), CXCL14/BRAK (6 out of 9), IL-8/CXCL8 (6 out of 9), and Prolactin (5 out of 9). In contrast, we saw increases in CXCL5/ENA-78 (6 out of 9).

### Drug effects on cytokine, chemokine, and growth factor profiles were observed across multiple cell lines

We also evaluated several drugs that belonged to additional classes of small-molecules (Supplementary Figure 1). First, we looked at GSK-3 inhibitor 9-ING-41 and saw decreases in VEGF (3 out of 3), CXCL9/MIG (3 out of 3), and CCL3/MIP-1 alpha (2 out of 3). Meanwhile, we observed increases in CXCL14 (3 out of 3), IL-8/CXCL8 (2 out of 3), sTRAIL-R2 (3 out of 3), and sTRAIL-R3 (2 out of 3). Next, we focused on Crizotinib, a c-MET inhibitor, and saw decreases in VEGF (3 out of 3), CXCL9/MIG (2 out of 3), and CXCL13/BLC/BCA-1 (2 out of 3). In contrast, we observed increases in IL-8/CXCL8 (2 out of 3), sTRAIL-R2 (2 out of 3), Prolactin (2 out of 3), and CXCL14 (2 out of 3). Next, we examined Dasatinib, a tyrosine kinase inhibitor, and observed decreases in IL-8/CXCL8 (3 out of 3), VEGF (2 out of 3), CXCL9/MIG, and CXCL5 (2 out of 3). In contrast, we noted increases in sTRAIL-R2 (3 out of 3), CXCL14/BRAK (3 out of 3), and Prolactin (2 out of 3). We next examined Duvelisib, a PI3K inhibitor and saw decreases in CCL3/MIP-1 alpha (2 out of 3), VEGF (3 out of 3), sTRAIL-R2 (2 out of 3), IL-8/CXCL8 (2 out of 3), CXCL9/MIG (2 out of 3) and CXCL14/BRAK (2 out of 3). We observed an increase in Prolactin (2 out of 3). We next analyzed Larotrectinib, a TRK inhibitor, and noted decreases in VEGF (3 out of 3), CXCL9/MIG (3 out of 3), CCL3/MIP-1 alpha (2 out of 3), and IL-8/CXCL8 (2 out of 3). Meanwhile, we observed increases in CCL5/RANTES (2 out of 3), IL-18/IL-1F4 (2 out of 3), and Prolactin (2 out of 3). We also analyzed Regorafenib, a multikinase inhibitor, and again noted decreases in VEGF (3 out of 3), CXCL9/MIG (3 out of 3), IL-8/CXCL8 (3 out of 3), CCL3 (2 out of 3), and CXCL5/ENA-78 (2 out of 3). In contrast, we noted increases in CXCL14/BRAK (2 out of 3), and Prolactin (2 out of 3). We then examined RET inhibitor Selpercatinib, and observed decreases in VEGF (3 out of 3), CXCL14/BRAK (2 out of 3), CCL3/MIP-1 alpha (2 out of 3), and sTRAIL-R2 (2 out of 3). Lastly, we saw increases in IL-8/CXCL8 (2 out of 3), CXCL5/ENA-78 (3 out of 3), and Prolactin (2 out of 3).

## DISCUSSION

The most commonly downregulated analyte in response to all treatment conditions was vascular endothelial growth factor (VEGF). VEGF is an angiogenic factor that is upregulated in many cancer types, including CRC, and promotes tumor angiogenesis. In CRC, VEGF expression in tumor tissue and patient plasma samples correlates with disease progression and metastasis [27]. Moreover, VEGF-positive tumors [28], high post-operative plasma VEGF concentrations [29], and high serum VEGF levels are correlated with decreased overall survival in CRC [30]. The downregulation of VEGF that we observed as a common trend despite heterogenous cell line mutational profiles and therapeutic mechanisms of action could suggest the possibility of off-target or non-specific effects. The identification of VEGF as a tumor cell-secreted marker that is commonly altered by small-molecules will require further interrogation. Another commonly downregulated analyte was CXCL9/MIG, an important chemokine for both recruitment and activation of leukocytes mediated by binding to CXCR3, a receptor expressed on activated T cells. It has been shown that expression of CXCL9/MIG is higher in patients with colon cancer as compared to healthy controls [31]. Furthermore, CXCL9/MIG expression was corelated with the presence of tumor-infiltrating lymphocytes as well as post-operative survival [31]. Next, we saw recurrences in IL-8/CXCL8 down-regulation post-treatment with our drug panel. IL-8/CXCL8 expression is significantly associated with colorectal tumorigenesis and metastasis [32] and has been suggested as a therapeutic target for this reason. Moreover, it is known that IL-8/CXCL8 induces epithelial-mesenchymal transition (EMT) in tumor cells via the PI3K/Akt signaling axis [33]. Another chemokine commonly downregulated was CCL3/MIP-1 alpha, which plays an important role in lymphocyte recruitment, activation, proliferation, and differentiation in colon cancer murine models [34]. Lastly, we observed a common post-treatment decrease in CXCL14/BRAK, a small chemokine with controversial effects in tumorigenesis [35–37]. The clinical correlation of this biomarker with disease prognosis remains unclear at this time, as several have reported that elevated levels of CXCL14/BRAK expression in tumor sections correlates with worse overall survival [38], yet others have reported the opposite [39].

Interestingly, CXCL14/BRAK was also among one of the analytes most commonly upregulated. We also noted a common increase in the hormone prolactin, which is commonly overexpressed in patients with colorectal cancer [40]. Next, we observed a recurrent upregulation of CCL5/RANTES which is chemotactic for many leukocytes and plays an important role in immune cell recruitment to inflammatory sites. In contrast, the CCR5/CCL5 axis has also been reported to play a role in the proliferation, metastasis, and formation of an immunosuppressive microenvironment [41]. Tumor-derived CCL5/RANTES has been shown to enhance regulatory T cell-mediated killing of cytotoxic T cells in colon cancer [42]. Moreover, CCL-5 deficiency has been shown to increase tumor infiltrating CD8+ T cells in the context of CRC [43]. Lastly, we noted an upregulation of CXCL5/ENA-78 under many of the treatment conditions across several cell lines, which may induce colorectal cancer angiogenesis [44].

We also monitored soluble receptors TNF-related apoptosis-inducing ligand Receptor 2 (sTRAIL-R2)/ Death Receptor 5 (sDR5) and TNF-related apoptosis-inducing ligand Receptor 3 (sTRAIL-R3). TRAIL-R2 is well-known as a cell surface receptor that triggers apoptosis upon binding with its cognate ligand, TNF-related apoptosis-inducing ligand (TRAIL). In contrast, TRAIL-R3 is known as a decoy receptor for TRAIL, as it lacks a cytoplasmic death domain rendering it unable to induce apoptosis. The soluble versions of these receptors presumably both function as decoy receptors that can bind and prevent TRAIL-mediated apoptosis. To our knowledge, soluble TRAIL receptors have not yet been characterized as potential biomarkers of immune response to therapeutics in the context of cancer. These may be novel biomarkers for assessing the innate immune system as impacted by cancer therapeutics and would be especially relevant in the context of immunotherapies such as αPD-1, αPD-L1, and αCTLA4. Furthermore, these are relevant biomarkers in the context of TRAIL-receptor agonists such as ABBV-621, IGN-8444, INBRX-109, and AMG-655 which could be bound by sTRAIL-R2, potentially reducing therapeutic efficacy. However, the extent to which soluble TRAIL-R2/R3 can predict therapeutic efficacy in humans or in mice remains to be determined.

We also analyzed soluble receptor ligand programed-death ligand 1 (sPD-L1). PD-L1 is a transmembrane molecule that belongs to the B7 family and acts by binding to PD-1 on the surface of lymphocytes to inhibit the differentiation and proliferation of immune cells [45]. In CRC, elevated expression of PD-L1 is associated with poor prognosis, survival, and lymph node metastasis [45]. Furthermore, PD-L1 expression due to IFN-γ signaling predicts poor survival in CRC [46]. PD-L1 is thought to be most relevant as a biomarker in the context of immunotherapy, where many have described both predictive and prognostic roles of PD-L1 in colorectal cancer, and several other cancer types [47]. PD-L1 is currently being evaluated as a biomarker of poor prognosis in patients with CRC undergoing immunotherapy [47]. The soluble version of PD-L1, specifically, is an emerging biomarker of focus in CRC and increased sPD-L1 expression post-neoadjuvant chemoradiotherapy is correlated with worse disease-free survival [9].

We observed heterogeneity in cytokine, chemokine, and growth factor responses across cell lines and across drug treatments. When the results were grouped by either (1) analytes that are correlated with immunosuppression or unfavorable prognosis or by (2) analytes that are correlated with immunostimulation or favorable prognosis, we found that it was difficult to make clear predictions about which combinations of therapeutics would promote anti-tumor immunity in the context of CRC. This heterogeneous response to therapeutic treatment indicates that therapeutic immunomodulation is not necessarily predictable based on the tumor cytokinome alone. However, our dataset is limited and perhaps clearer trends would emerge with a greater sample size. Moreover, this dataset is amenable to building a larger database with other cytokinome data. We present our results as a novel platform with a large panel of relevant cytokines, chemokines, and growth factors that can impact therapeutic and immune response in the complex tumor microenvironment. Future studies can focus on specific cell lines, tumor types, classes of drugs, and subsets of cytokines. Additionally, we are currently pursuing the utilization of this platform for the investigation of drug combinations.

## MATERIALS AND METHODS

### Cell culture

Human colorectal cancer cells HCT-116, HT-29, and KM12C were used in this study. HCT-116 and HT-29 were cultured in McCoy’s 5A (modified) Medium supplemented with 10% FBS and 1% Penicillin-Streptomycin. KM12C cells were cultured in Eagle’s Minimal Essential Medium Supplemented with 10% FBS and 1% Penicillin-Streptomycin. All cell lines were incubated at 37°C in a humidified atmosphere containing 5% CO_2_. Cell lines were authenticated and tested to ensure the cultures were free of mycoplasma infection.

### Measurement of cell viability

Cells were plated at a density of 3 × 10^3^ cells per well in a 96-well plate (Greiner Bio-One, Monroe, NC, USA). Cell viability was assessed using the CellTiter Glo assay (Promega, Madison, WI, USA). Cells were mixed with 25 μl of CellTiter-Glo reagents in 100 μl of culture volume, and bioluminescence imaging was measured using the Xenogen IVIS imager (Caliper Life Sciences, Waltham, MA, USA).

### Collection of culture supernatants used in cytokine measurements

Cells were plated at 3.5 × 10^4^ cells in a 48 well plate (Thermo Fisher Scientific, Waltham, MA, USA) in complete medium and incubated at 37°C with 5% CO_2_. At 24 hours after plating, almost all the tumor cells were adherent to the bottom of the flask and the complete medium was replaced with drug-containing medium. Subsequently, the culture supernatants were collected after 48 hours of incubation and were frozen at –80°C until the measurement of cytokines was performed. The day of analysis, samples were thawed and centrifuged to remove cellular debris.

### Cytokine, chemokine, and growth factor profiling

An R&D systems Human Premixed Multi-Analyte Kit (R&D Systems, Inc., Minneapolis, MN) was run on a Luminex 200 Instrument (LX200-XPON-RUO, Luminex Corporation, Austin, TX) according to the manufacturer’s instructions. Cell culture supernatant levels of TNF-alpha, IL-6, IL-8/CXCL8, Ferritin, IFN-beta, IL-10, CCL2/JE/MCP-1, VEGF, CXCL13/BLC/BCA-1, IFN-gamma, CCL20/MIP-3 alpha, CCL3/MIP-1 alpha, CCL22/MDC, CCL4/MIP-1 beta, IL-4, IL-17/IL-17a, TRAIL R2/TNFRSF10B, GM-CSF, CXCL5/ENA-78, CXCL9/MIG, G-CSF, CXCL11/I-TAC, Granzyme B, CCL5/RANTES, Prolactin, IFN-alpha, CXCL14/BRAK, IL-12/IL-23 p40, CXCL10/IP-10/CRG2, CCL7/MCP-3/MARC, IL-7, CCL8/MCP-2, TRANCE/TNFSF11/RANK L, IL-15, TRAIL R3/TNFRSF10C, CCL11/Eotaxin, IL-18/IL-1F4, TRAIL/TNFSF10, IL-21, and C-Reactive Protein/CRP were measured.

### Bioinformatics analysis

A quantitative analysis with 6 standards and a minimum of 50 counts per bead region was used with the Luminex to generate analyte values reported as picograms/ milliliter (pg/mL). Cytokine concentrations less than the lower limit of detection for each particular cytokine were recoded as zero. Cytokines without detectable expression levels were removed from further analysis of each cell line. Each drug was tested at different inhibitory concentrations (IC-10, IC-30, IC-50, IC-70, and IC-90), and these varying concentrations were used to measure a dose-response effect on cytokine expression. Cytokine dose-response effect was modeled by simple linear regression for each drug. The slopes of the linear regressions were compared. Data analysis and visualization were generated using R (R Development Core Team, 2020).

## SUPPLEMENTARY MATERIALS






