# Supplementary material for: A high-throughput customized cytokinome screen of colon cancer cell responses to small-molecule oncology drugs
Source: Oncotarget. 2021 Sep 28;12(20):1980–91. doi: 10.18632/oncotarget.28079 (PMC8487726; doi:10.18632/oncotarget.28079)
Supplement: Supplementary file 1 [file oncotarget-12-1980-s001.pdf]

A high-throughput customized cytokinome screen of colon cancer cell responses to small-molecule oncology drugs

SUPPLEMENTARY MATERIALS

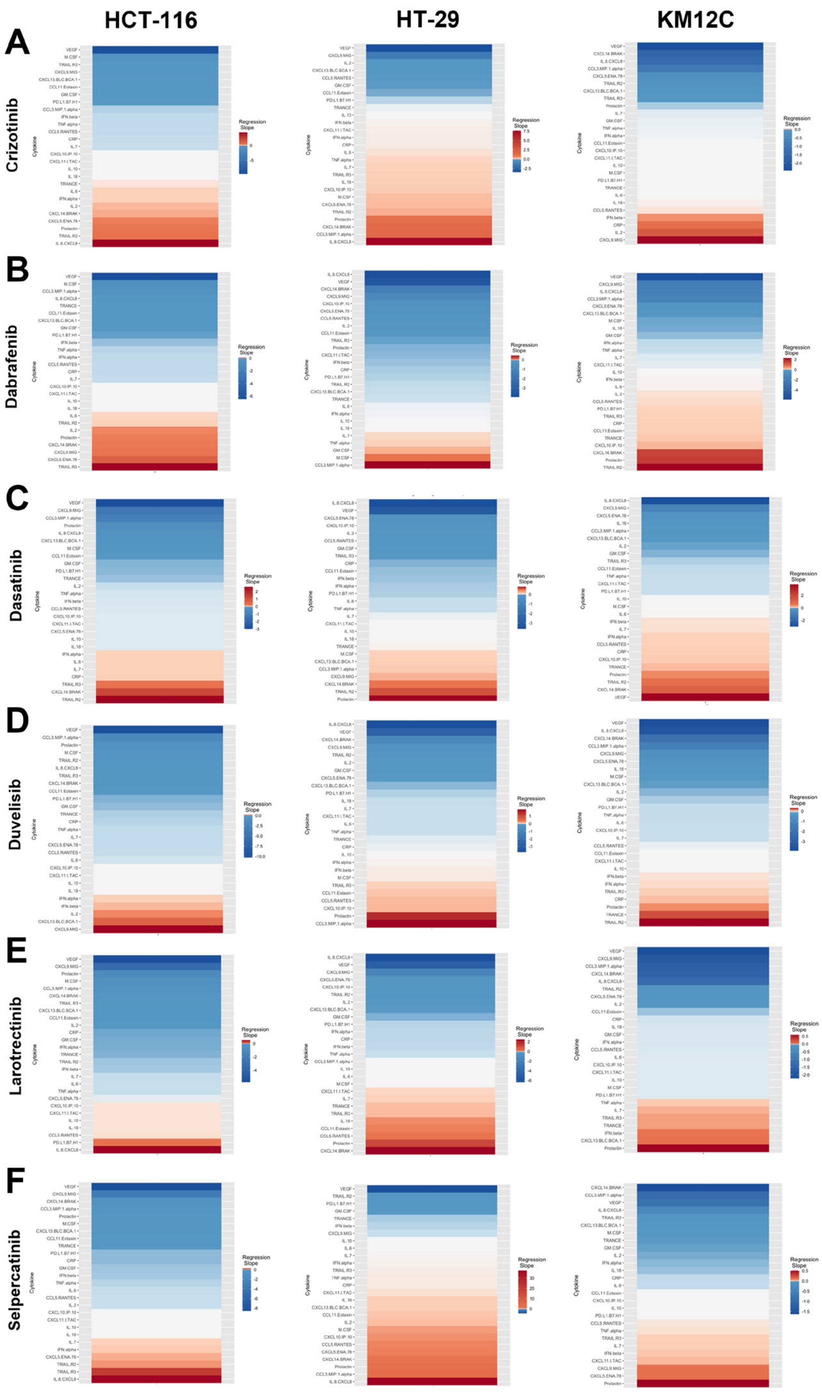

Supplementary Figure 1: Heatmaps displaying regression slopes of cytokine profiles. (**A**) Heat maps based on regression slopes for HCT-116, HT-29, and KM12C after 48-hour treatment of increasing doses of Crizotinib, (**B**) Dabrafenib, (**C**) Dasatinib, (**D**) Duvelisib, (**E**) Larotrectinib, or (**F**) Selpercatinib.

Supplementary Table 1: The five most significantly upregulated and downregulated analytes in response to therapeutic treatment. See Supplementary Table 1

Supplementary Table 2: IC-50 values for selected cell lines and small molecules. See Supplementary Table 2
